# Pregnancy Arrhythmias: Management in the Emergency Department and Critical Care

**DOI:** 10.3390/jcm13041095

**Published:** 2024-02-15

**Authors:** Elena Conti, Nunzio Dario Cascio, Patrizia Paluan, Giulia Racca, Yaroslava Longhitano, Gabriele Savioli, Manfredi Tesauro, Roberto Leo, Fabrizio Racca, Christian Zanza

**Affiliations:** 1 Division of Anesthesia and Critical Care Medicine, Azienda Ospedaliera Ordine Mauriziano, 10128 Turin, Italy; econti@mauriziano.it (E.C.); ncascio@mauriziano.it (N.D.C.); ppaluan@mauriziano.it (P.P.); fracca7766@gmail.com (F.R.); 2 Department of Anesthesiology and Perioperative Medicine, University of Pittsburgh, Pittsburgh, PA 15260, USA; 3 Department of Emergency Medicine—Emergency Medicine Residency Program, Humanitas University-Research Hospital, 20089 Rozzano, Italy; 4 Emergency Medicine and Surgery, IRCCS Fondazione Policlinico San Matteo, 27100 Pavia, Italy; 5 Geriatric Medicine Residency Program, University of Rome “Tor Vergata”, 00133 Rome, Italy; 6 Department of Systems Medicine, University of Rome “Tor Vergata”, 00133 Rome, Italy

**Keywords:** pregnancy, arrhythmias, structural heart disease

## Abstract

Pregnancy is closely associated with an elevated risk of arrhythmias, constituting the predominant cardiovascular complication during this period. Pregnancy may induce the exacerbation of previously controlled arrhythmias and, in some instances, arrhythmias may present for the first time in pregnancy. The most important proarrhythmic mechanisms during pregnancy are the atrial and ventricular stretching, coupled with increased sympathetic activity. Notably, arrhythmias, particularly those originating in the ventricles, heighten the likelihood of syncope, increasing the potential for sudden cardiac death. The effective management of arrhythmias during the peripartum period requires a comprehensive, multidisciplinary approach from the prepartum to the postpartum period. The administration of antiarrhythmic drugs during pregnancy necessitates meticulous attention to potential alterations in pharmacokinetics attributable to maternal physiological changes, as well as the potential for fetal adverse effects. Electric cardioversion is a safe and effective intervention during pregnancy and should be performed immediately in patients with hemodynamic instability. This review discusses the pathophysiology of arrythmias in pregnancy and their management.

## 1. Introduction

Disturbances of heart rhythm are the most common cardiovascular complication of pregnancy [1]. Over the past 20 years, prevalence of arrhythmias in pregnancy has risen in the United States [2]. Hospitalizations due to arrhythmias in pregnancy have increased by 58% from 2000 to 2012, mainly due to a rise in atrial fibrillation and in ventricular tachycardia [3]. In a comprehensive 10-year retrospective analysis of maternal cardiovascular deaths, arrhythmias emerged as a significant contributor, being identified as the immediate or underlying cause in 10.7% of cases [4]. Women 41–50 years of age, or with cardiovascular disease (like congenital heart disease) or cardiovascular comorbidities (i.e., hypertension, diabetes mellitus, and obesity) more frequently experience arrhythmias [4,5,6,7].

Arrhythmias can occur in pregnancy for the first time, but pregnancy can also worsen a previously controlled arrhythmia due to its physiological changes [1]. Particularly noteworthy is the heightened risk associated with arrhythmias of ventricular origin, which not only elevate the likelihood of syncope but also substantially augment the probability of sudden cardiac death [7,8].

Pregnancy itself carries an increased risk of arrhythmias, because of proarrhythmic mechanisms caused by cardiovascular, autonomic, and hormonal changes [2]. The postpartum period of adaptive changes in the circulatory system is the most arrhythmogenic period [9,10]. This underscores the critical importance of understanding and managing arrhythmias during pregnancy, emphasizing the need for a multidisciplinary approach, vigilant monitoring, and timely intervention to safeguard maternal health and ensure optimal fetal outcomes.

This review discusses the pathophysiology of arrythmias in pregnancy and their management.

## 2. Pathophysiology of Arrythmias in Pregnancy

**Figure 1:**
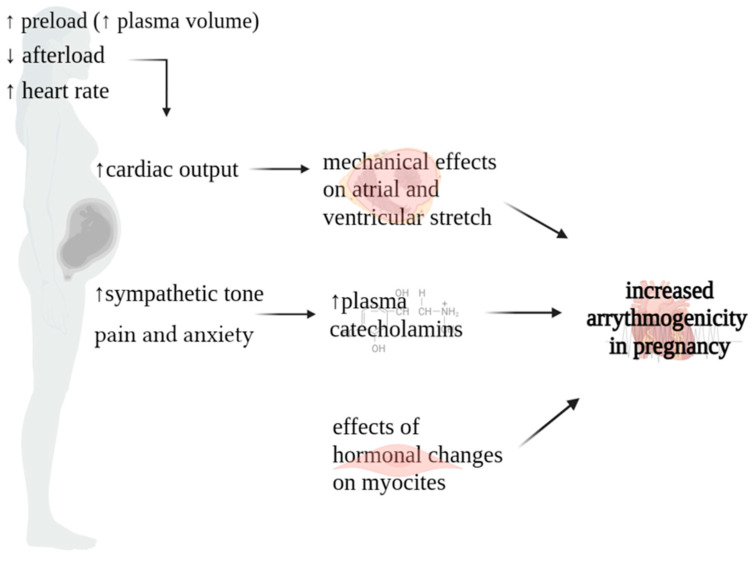
Physiopathology of arrythmias in pregnancy.

Maternal hemodynamics undergo profound changes throughout pregnancy, with significant alterations initiating shortly after conception, peaking during the second and early third trimesters, and maintaining relative stability until the onset of labor and delivery. These hemodynamic shifts play a crucial role in supporting the developing fetus and adapting to the evolving demands of pregnancy. The heightened susceptibility to arrhythmic events in pregnant women results from a complex interplay of autonomic, hormonal, and cardiovascular modifications (Figure 1) [2]. Specifically, heightened levels of plasma catecholamines, amplified ventricular end-diastolic volume resulting from intravascular volume expansion, mechanical effect of atrial stretch, and the multifaceted influence of hormonal and emotional changes collectively promote a proarrhythmic environment [11,12].

Cardiac output (CO) increases throughout pregnancy. It experiences a surge as early as six weeks into gestation, persisting until 20–24 weeks, peaking at levels 30–50% higher than the non-pregnant baseline. In instances of multiple gestation pregnancies, this augmentation is further pronounced, with CO escalating by 60–70% [13,14]. This surge is influenced by changes in three key factors governing CO: (a) augmented preload stemming from the rise in plasma volume; (b) diminished afterload attributable to the decline in systemic vascular resistance (SVR); (c) elevated heart rate (HR) [15]. During the early stages of gestation, plasma volume undergoes a gradual expansion of 10–15%, intensifying its augmentation to 40–50% above pre-pregnancy levels by the 30th–34th weeks. The concurrent reduction in systemic vascular resistance (SVR) can be attributed to a combination of factors. Firstly, the uteroplacental circulation establishes a low-resistance circuit. Secondly, estrogen-induced vasodilatation plays a crucial role, with estrogen promoting nitric oxide production [16,17]. Finally, during the first trimester, there is an elevation in resting HR, exhibiting an average escalation of 10–30 beats per minute (bpm). This upward trend persists, progressively intensifying, until reaching its zenith at 34 weeks, with a maximum HR of 91 bpm (3rd to 97th centiles: 68–115 bpm) [18]. Subsequently, at 40 weeks, a marginal decrease is noted, with the median HR settling at 89 bpm (3rd to 97th centiles: 65–114 bpm). This increase in HR is likely due to hormonal changes in the early stages of pregnancy, while the later increase is linked to augmented left atrial diameter and sympathetic activation [19].

The rise in cardiac output contributes to optimal fetal growth and development. However, in patients with underlying heart disease, this increase in cardiac output can cause cardiac failure during the latter half of pregnancy. In addition, these hemodynamic changes lead to myocardial atrial and ventricular stretching, which results in activation of stretch-sensitive ion channels, with membrane depolarization, shortened refractoriness, slowed conduction, and spatial dispersion of refractoriness, resulting in potential arrhythmogenesis [20,21].

Another cause of arrhythmogenicity during pregnancy is the change in sympathetic tone. In a physiological context, pregnancy is characterized by a decrease in parasympathetic activity and an increase in sympathetic activity during periods of rest. This heightened sympathomimetic tone is influenced by various factors, encompassing neurohormonal alterations throughout pregnancy and heightened sympathetic responses triggered by pain and anxiety during labor and delivery [14,22]. Increased sympathetic activity may contribute to abnormal automaticity or reentry activity [23,24,25].

Concerning hormonal changes, cardiac myocytes have estrogen and progesterone receptors. The downstream effects of estrogen and progesterone on cardiac myocytes are not well understood, but studies have shown these hormones play a role in repolarization [22]. Both animal and human studies have described the arrhythmogenic potential of estrogen and progesterone by increasing the number and responsiveness of adrenergic receptors within the myocardium [26,27].

## 3. Echocardiographic and Electrocardiographic Changes during Pregnancy

### 3.1. Echocardiographic Changes

The most important echocardiographic modifications associated with pregnancy are predominantly attributed to pregnancy-induced hypervolemia and encompass the following aspects: (a) left atrial size increases by 0.4–0.5 cm, while the left ventricular diastolic dimension expands by 0.2–0.4 cm; (b) left ventricular mass experiences a rise of 5–10%, resulting in eccentric hypertrophy; (c) ventricular global systolic function shows no significant alteration; however, global longitudinal strain decreases to the lower end of the normal range in the later stages of pregnancy, maintaining stability until term; (d) each valve may exhibit mild regurgitation, especially in the third trimester; (e) small pericardial effusions are prevalent, reported in up to 25–40% of normal pregnancies; (f) slight elevations in pulmonary arterial pressure are observed [28,29,30,31,32].

These changes typically resolve three to six months postpartum.

### 3.2. Electrocardiographic Changes

Anatomic and physiologic changes of the heart and chest wall during pregnancy may cause alterations also in the electrocardiogram [2,3,4,5,6,7,8,9,10,11,12,13,14,15,16,17,18,19,20,21,22,23,24,25,26,27,28,29,30,31,32,33,34]. All these pregnancy-related ECG effects usually restore following delivery. The principal alterations are described below.

During pregnancy, sinus tachycardia is common. The heart rate increases by 10–20 beats per minute and the upper limit of the resting HR typically is not greater than 100 bpm [18].

The heart is rotated toward the left, resulting in a 15–20 degree left axis deviation. As a consequence, leftward shift in the QRS axis may be seen.

Other findings include shortened PR interval, increased R/S ratio in leads V1 and V2, Q waves and inverted T waves in the inferior leads, and nonspecific transient ST-T changes. In addition, a QRS prolongation, due to ventricular dilatation, may be also found in pregnancy.

The uncorrected QT shortens in tachycardia. However, the QTc interval is longer in the second and third trimester of pregnancy compared with non-pregnancy, although it is still within normal range [35,36,37]. A recent study reported that QTc intervals in women in the 1st, 2nd, and 3rd trimester of pregnancy in the puerperium are respectively 420.57 (SD 24.91), 427.58 (SD 18.61), 426.56 (SD 16.12), and 428.83 (SD 22.52) seconds [35].

With regard to T-peak to T-end interval (TpTe interval), an increase in his duration may be observed starting in the first trimester with highest values observed in the postpartum period [35]. The TpTe interval is the distance between the T-wave peak point and the returning point to the isoelectric line. The electrocardiographic TpTe interval is considered to be a more sensitive diagnostic marker of arrhythmogenesis, compared to the traditionally used QT interval, especially with the accompanying change in the shape of the T wave to biphasic [38]. In particular, TpTe interval prolongation (i.e., over 120 ms) may be associated with development of potentially lethal ventricular arrhythmia and polymorphic ventricular tachycardia.

Therefore, for women with an inherent predisposition to repolarization abnormalities, pregnancy may constitute a phase of heightened susceptibility to cardiac arrhythmias. Maximum QTc and T-peak to T-end intervals are indicators of sympathetic activation [39]. In addition, the duration of the TpTe interval correlates also with the thickness of the left ventricular wall [40,41].

## 4. Pharmacotherapy and Cardiological Procedures during Pregnancy and Lactation

The management of arrhythmias in pregnant women largely parallels that in non-pregnant patients, with minor adjustments mandated by considerations for fetal safety. Nevertheless, in case of hemodynamically significant arrythmias, the primary objective shifts to the prompt restoration of normal hemodynamics [42].

The biggest concern associated with the administration of antiarrhythmic drugs (AADs) during pregnancy is the potential for adverse fetal side effects and teratogenicity, especially in the first trimester when organogenesis begins. Moreover, the use of AADs in pregnancy requires attention to potential changes in maternal pharmacokinetics, such as an increase in intravascular volume [43,44]. Lastly, during lactation, special consideration should be given to medications that may adversely affect the newborn. While some medications are safe in pregnancy, their metabolism and concentration in breast milk can be of concern during lactation. One example of this is the beta-blocker nadolol, which has a high concentration in breast milk [42]. As a consequence, the discussion of medications during breastfeeding should include consideration of the underlying conditions of the pregnant patient, the optimal treatment for their arrhythmia, and whether there is a reasonable alternative that has similar efficacy but is safer for breastfeeding. If there are no medication alternatives that are efficacious for the patient and safe with lactation, lactation may need to be avoided or monitored closely for potential side effects (e.g., excess bradycardia in the case of nadolol). This decision should be based on a shared decision-making discussion with the patient and family that considers the negative impact of deferring the recommended pharmacological therapy on maternal health in the postpartum period balanced against the importance of breastfeeding to the postpartum patient and baby [42].

In 1979, the Food and Drug Administration (FDA) established five letter risk categories (i.e., A, B, C, D, or X) to indicate the potential of a drug to cause birth defects if used during pregnancy. Most antiarrhythmic drugs are Class C or D. In this classification system, drugs falling into Class X are strictly contraindicated during pregnancy. In contrast, Class A drugs have demonstrated no fetal risk in controlled studies. Class B encompasses drugs that exhibit no apparent risk to the human fetus based on available data observed in animal studies, although comprehensive human studies are currently lacking. Drugs categorized as Class C present a scenario where there are limited data available regarding their use in human pregnancy. Nevertheless, these drugs have been investigated in animal reproduction studies, revealing adverse fetal effects. Moving to Class D, this class is reserved for drugs that have been demonstrated to induce adverse effects on the fetus when administered during pregnancy in humans. Several authors had replaced the FDA rating for drugs in pregnancy by a narrative risk [2]. Since randomized clinical trials evaluating the effects of AADs in pregnancy are lacking, risk versus benefit must be always considered, and careful assessment of efficacy and safety should be performed before initiation. Lastly, the lowest effective dose should be used [2].

Antiarrhythmic drugs and their safety profile and adverse events in pregnancy and lactation are summarized in Table 1.

### 4.1. Beta-Blockers (FDA Class C)

Beta-blockers have been widely used during pregnancy. These medications cross the placenta, and long-term treatment is associated with a small risk of intrauterine growth restriction (IUGR), preterm birth, neonatal hypoglycemia, bradycardia, and hypotension [45,46]. Most of the studies on maternal beta-blocker therapy are based on pregnant patients with hypertensive disorders of pregnancy, where fetal growth could be affected by the underlying condition and not necessarily the drug itself [47]. Beta1 selective beta-blockers are associated with lower rates of IUGR and decreased effects on uterine activity and peripheral vasodilation. Nonselective beta-blockers are associated with higher rates of IUGR [1].

Propranolol and metoprolol are the preferred beta-blockers during pregnancy [46,48,49,50,51]. In particular, metoprolol is associated with the smallest reduction in birth weight [52], and they both may increase uterine tone [42]. Nadolol has also been safely used in pregnancy. Atenolol is the only beta-blocker listed in FDA Class D due to increased risk of congenital malformations [53,54,55,56,57,58]. Atenolol and nadolol may be excreted at higher levels in breast milk; therefore, they are not recommended during lactation.

### 4.2. Calcium Channel Blockers (FDA Class C)

Calcium channel blockers (CCB) have not been associated with increased risk of congenital malformation [59,60]. Due to the mechanism of action, CCBs may cause maternal hypotension, fetal bradycardia, and tocolysis. Prior studies suggested an increased risk of neonatal seizures with CCB use in the third trimester; however, this was not shown in a recent large cohort study [61]. Verapamil had no significant risk of teratogenicity; minimal maternal hypotension and fetal bradycardia have been described up to 10 mg intravenously. Diltiazem clinical use is controversial due to fetal adverse effects noted in animal models [62,63,64]. In the short term, CCB should be avoided and adenosine can be used. Both verapamil and diltiazem are safe during the lactation [65].

### 4.3. Class IA AADs (FDA Class C)

*Quinidine* and *procainamide* could cause maternal arrhythmias like torsade de pointes without teratogenic effects. Thrombocytopenia with quinidine and drug-induced lupus with procainamide are the common side effects. Both drugs are compatible with lactation but with caution and for short-term use.

Quinidine has a long track record of safety with only rare reported fetal adverse effects. In addition, mild uterine contractions, premature labor, neonatal thrombocytopenia, and cranial nerve VIII damage have been rarely reported with low-quality evidence.

Regarding procainamide, limited data in pregnancy are reported [66,67].

### 4.4. Class IB AADs

*Lidocaine* (FDA Class B) has a safety profile in pregnancy, crosses the placenta and can be used during lactation; in mice, a therapeutic dose range had no effects on uteroplacental circulation, amniotic fluid pressure, or fetal heart rate [68,69]. In the first three months, lidocaine exposure was not associated with an increased risk of birth defects or adverse events, with an overall normal perinatal course [67].

### 4.5. Class IC AADs (FDA Class C)

*Flecainide* is a sodium channel blocker used in the treatment of supraventricular tachycardia, atrial arrhythmias, and CPVT. It should not be used in patients with coronary artery disease or structural heart disease [70]. In maternal and fetal arrhythmias, flecainide can safely be used despite it being found in breast milk [2,71]. The starting dose (300 mg/day) is generally considered safe and it is free from teratogenic effects [72]. The literature reports only delayed sternal and vertebral ossification observed in rats when used at very high doses [2].

In one single case report, no neonatal adverse outcomes were reported in terms of Wolff–Parkinson–White syndrome, SVT, and premature ventricular beats [2].

### 4.6. Class III AADs

Among Class III agents, *sotalol* (FDA Class C), a potassium channel blocker with beta-blocker properties, is considered safe during pregnancy and lactation, with only two low-risk side effects, fetal bradycardia and hypoglycemia, and without teratogenic effects in animal models [71]. Due to its QT-prolonging effects, there is risk of torsade de pointes [1]. In pregnancy, the pharmacokinetics are not significantly altered, although it is more rapidly cleared after intravenous administration during pregnancy [73]. It is compatible with lactation, but caution is required.

*Amiodarone* (Food and Drug Administration Class D) should be used only for refractory and/or life-threatening arrhythmias, because its effects on the fetus are independent of dose and duration. Adverse fetal effects include fetal hypothyroidism with congenital goiter, growth retardation, prematurity, neurodevelopmental abnormalities, and preterm birth [1,21]. If there are no other options, it must be used for as short a time as possible. Amiodarone must be avoided during lactation.

*Dronedarone* (FDA Class X) should not be used in pregnancy because of teratogenic effects such as vascular and limb abnormalities and cleft palate [2]. Its use during lactation is also contraindicated.

Data on *dofetilide* (FDA Class C) in pregnancy are lacking. Bradycardia and skeletal abnormalities have been observed in animal models [74,75].

Data on *ibutilide* (FDA Class C) are restricted to a few case reports for atrial flutter/AF cardioversion; thus, it should be used with caution in pregnancy and lactation. No adverse fetal effects were reported. In these case reports, mothers received pre-treatment with magnesium [76,77].

### 4.7. Other Antiarrhythmic Drugs

Due to its short half-life, *Adenosine* (FDA Class C) is the preferred medication to terminate maternal SVT in pregnancy; moreover, it is also safe during lactation. Starting dose should be 6–12 mg and pregnancy alters its metabolism because the biochemical velocity of adenosine deaminase is slowed down, and the resulting hypotension is countered by intravascular volume increasing [2,78,79,80,81].

*Digoxin* (FDA Class C) is safe in pregnancy and its concentrations are similar both in the mother and newborn [82,83,84]. Both blood levels and clinical signs of digoxin toxicity must be assessed because digoxin-like fragments could result in false positives in blood tests [85], although digoxin is excreted in trace into breast milk without relevant newborn side effects [82,83,84,85,86].

*Ivabradine* is contraindicated in pregnancy and lactation due to the risk of fetal growth retardation and neonatal hemodynamic impairment.

### 4.8. Electrical Synchronized Cardioversion

The safety and efficacy of cardioversion in pregnancy have been well-established, with a particular emphasis on immediate intervention for patients experiencing hemodynamic instability or cases where rate control measures are unsuccessful [2,87,88]. Obliviously, defibrillation pads should be strategically placed away from the gravid uterus and must not be placed on breast tissue. Although cardioversion itself does not compromise fetal blood flow, it may induce uterine contractions, posing a theoretical risk of preterm labor [62,88]. This underscores the importance of maintaining appropriate facilities for emergency caesarean section during direct current cardioversion procedures in pregnant women. While the risk of inducing fetal arrhythmias is minimal [2], it is advisable to conduct fetal monitoring due to the amniotic fluid’s conductivity for elective cardioversion, not for emergency defibrillation or cardioversion [89].

Various studies have reported successful cardioversion outcomes with energies ranging from 50–400 J, demonstrating success rates exceeding 90% without adverse effects on the fetus [21,90].

### 4.9. Electrophysiology Procedures

*Catheter ablation*, a therapeutic intervention for refractory and/or life-threatening arrhythmias, has been demonstrated to be safe during pregnancy, although the preference is to defer the procedure to the postpartum period when feasible [71,91]. In cases where postponement is not an option, catheter ablations are cautiously performed during the second trimester, utilizing echocardiographic guidance to minimize or eliminate radiation exposure [1,2].

Radiation exposure during pregnancy is a critical consideration, with most fetal effects occurring before 17 weeks of gestation at doses exceeding 200 mGy [92]. Exposure below 50 mGy has not been linked to fetal abnormalities [93]. Exposure to radiation from 8 to 15 weeks of gestation, at levels between 60 and 310 mGy, has been associated with a potential risk of mental retardation [94]. Although the lifetime risk of malignancy remains low, certain case-control studies have suggested that even minimal antenatal exposure, as low as 10 mGy, may increase the risk of childhood cancer [95].

For ablation procedures, patients should be positioned in the left lateral tilt after the second trimester to prevent aortocaval compression, and continuous fetal monitoring is essential [2].

The implantation of *cardiac-defibrillators (ICD) and pacemakers* is deemed safe during pregnancy, with devices implanted under echocardiographic guidance and minimal fluoroscopy, particularly for women with indications arising during pregnancy [2]. The presence of previously implanted pacemakers and ICDs do not elevate maternal or fetal risks. Most pregnancies involving pacemaker patients are uneventful from a pacemaker perspective [2] and ICD shocks have not shown adverse fetal effects [96].

The reprogramming of pacemaker rate response to accommodate the increasing heart rate demand during pregnancy is advisable. Skin irritation at the pacemaker site due to breast hypertrophy has been reported [2].

Asynchronous mode and using bipolar cautery during caesarean delivery is essential to avoid pacing inhibition caused by noise interference [97]. For ICDs following adequate cardiac monitoring, it is not mandatory to disable shock therapy during labor and delivery [2].

## 5. Management of Different Types of Arrhythmias in Pregnancy

The first consideration in addressing arrhythmias in pregnant women is that the overall approach is analogous to that in non-pregnant patients, with differences based in particular on fetal safeguard. Notably, instances of arrhythmias causing hemodynamic instability necessitate immediate electric cardioversion [42]. Before initiating long-term medical therapy, it is imperative to assess potential triggers for arrhythmias. These triggers encompass severe electrolyte abnormalities, illicit drug use, supplements, and specific obstetric medications such as terbutaline and magnesium sulfate [1].

A comprehensive summary of arrhythmia treatment is provided in Table 2.

### 5.1. Premature Beats

Premature atrial and ventricular beats are very common in pregnancy (i.e., 50–60% of pregnant patients). They may present with palpitations [1,33]. Premature ventricular or atrial ectopic beats generally resolve spontaneously after delivery [33].

Although premature beats are often benign, in some pregnant women they can be associated with structural heart disease. In particular, premature ventricular contractions (PVC) may be an initial presentation of a cardiomyopathy. As a consequence, further evaluation is prudent. Patients with preserved systolic function should be reassured [98]. Medical therapy for PVCs is indicated for significant symptoms and/or in the setting of a reduced left ventricular ejection fraction. First-line therapy with calcium channel blockers or beta-blockers, excluding atenolol, is recommended [98].

Additionally, frequent premature atrial contractions (>100 beats in 24 h) require further evaluation, because they have been shown to increase the risk of new-onset atrial fibrillation, supraventricular tachycardia, and cardiovascular morbidity and mortality [99,100].

### 5.2. Inappropriate Sinus Tachycardia

During pregnancy, there is a natural increase in heart rate by 10–20 beats per minute, yet the resting heart rate typically does not exceeds 95 beats per minute [101]. Inappropriate sinus tachycardia (IST) is characterized by an elevated resting heart rate, exceeding 100 beats per minute or maintaining an average heart rate above 90 beats per minute over a 24 h period, in the absence of secondary causes such as anemia, thyroid dysfunction, infections, illicit drug use, heart or pulmonary diseases. Symptoms of IST encompass palpitations, chest discomfort, fatigue, dizziness, and reduced exercise tolerance. Notably, published case reports suggest that IST is generally well-tolerated without adverse maternal or fetal outcomes [102,103].

### 5.3. Atrial Fibrillation and Atrial Flutter

Atrial fibrillation (AF) stands as the most prevalent arrhythmia during pregnancy, accounting for 27 per 100,000 pregnancy hospitalizations for arrhythmias [3]. The incidence of AF is notably higher in women with structural heart disease compared to those without structural heart disease [104]. Atrial flutter and AF are managed similarly, with limited available data on the prevalence of atrial flutter alone [2]. Risk factors contributing to an increased likelihood of AF include obesity, age older than 40, congenital heart disease, preexisting history of AF, beta-blocker use before pregnancy, and valvular heart disease [105,106]. AF and atrial flutter during pregnancy are associated with adverse maternal and fetal outcomes. Maternal complications encompass heart failure and thromboembolic events. Fetal complications include intrauterine growth restriction, intraventricular hemorrhage, respiratory distress syndrome, and a higher incidence of neonatal intensive care unit admissions [105,107]. Additionally, agents used for rate control may induce maternal hypotension and reduced placental perfusion, heightening the risk of preterm labor.

A new diagnosis of AF or atrial flutter should trigger a transthoracic echocardiogram to assess for structural heart disease. Furthermore, other potential causes such as thyroid disease, electrolyte abnormalities, pulmonary embolism, and alcohol abuse should be ruled out [108].

If the patient is hemodynamically unstable, immediate synchronized cardioversion of AF and atrial flutter is indicated [11,108]. Indeed, every condition characterized by hemodynamic deterioration may cause placental hypoperfusion. Fetal monitoring is recommended during and immediately after synchronized cardioversion.

If the patient maintains hemodynamic stability, the initial approach involves rate control with beta-blockers serving as a viable option for achieving this control. The combination of beta-blockers and digoxin may also be considered. Verapamil can be used, if necessary, although calcium channel blockers have less robust supportive data [33]. Electrical synchronized cardioversion is indicated in cases where rate control proves inadequate. It is imperative to position these patients in the left lateral tilt position to avoid aortocaval compression, especially after the second trimester of pregnancy.

In order to minimize the risk of stroke, electrical cardioversion should be administered within 48 h of AF onset. Transesophageal echocardiography (TEE) may be necessary if the onset of AF is unclear, to exclude intracardiac thrombus before cardioversion. Considering the risk of thromboembolism in pregnant patients with AF or atrial flutter, heparin compounds, particularly low-weight-molecular heparin, are the preferred anticoagulants [2]. If AF persists for more than 48 h, it should be managed with a minimum of 3 weeks of anticoagulation before cardioversion, unless TEE can rule out thrombus formation. Anticoagulation is advisable for at least 4 weeks after cardioversion in all women, unless an alternative indication for anticoagulation necessitates a more extended course.

In cases of recurrent or refractory AF requiring rhythm control, flecainide or sotalol can be considered [2]. While catheter ablation with minimal fluoroscopy is an option for refractory symptomatic cases, it is typically deferred until the postpartum period for overall safety and optimal outcomes [109,110].

### 5.4. Supraventricular Tachycardia

Supraventricular tachycardia (SVT) ranks as the second most common arrhythmia during pregnancy, occurring in 22 per 100,000 pregnancy hospitalizations [3]. The most common subtypes of SVT are atrioventricular nodal reentrant tachycardia (AVNRT) and atrioventricular reentrant tachycardia (AVRT). Approximately 20% of women with pre-existing SVT experience exacerbations during pregnancy, with SVT typically presenting in the second trimester [5]. Symptoms include sudden-onset palpitations, often accompanied by dyspnea, chest discomfort, or presyncope.

Patients with SVT, both AVNRT and AVRT are managed similarly.

If the patient is hemodynamically unstable, immediate synchronized cardioversion is indicated. If the patient is hemodynamically unstable for acute termination, vagal maneuvers, such as the Valsalva maneuver or carotid sinus massage, are the first-line therapy, followed by adenosine [78]. It is crucial to note that in supine patients, the uterus’s caval compression, common after 20 weeks of gestation, makes it imperative to perform vagal maneuvers by placing the patient in the left lateral tilt position to prevent aortocaval compression, especially after the second trimester of pregnancy.

For pregnant women with recurrent SVT, except for those with known pre-excitation or a history of Wolff–Parkinson–White syndrome, beta-blocker therapy is the first-line approach. The combination of beta-blockers with digoxin may be considered, and calcium channel blockers serve as second-line agents. Previous electrocardiograms in sinus rhythm should be assessed for preexcitation, and concern arises in women with pre-excited atrial fibrillation, which may degenerate into ventricular fibrillation [2]. In patients with evidence of preexcitation, atrioventricular nodal blockade alone should be used cautiously due to the risk of subsequent conduction over the accessory pathway, potentially placing the patient at risk of atrial fibrillation degenerating into ventricular arrhythmias. As a consequence, in these patients, beta-blockers must be used in conjunction with flecainide [111]. Digoxin is contraindicated for managing atrioventricular re-entrant tachycardia in the presence of pre-excitation on the resting electrocardiogram.

Upon a new supraventricular tachycardia (SVT) diagnosis, a transthoracic echocardiogram is advised to assess for structural heart disease.

Catheter ablation with minimal fluoroscopy can be considered in refractory cases [111,112], but it is generally preferred to defer ablation until the postpartum period [2].

### 5.5. Ventricular Arrhythmias

While ventricular arrhythmias (VAs) are rare during pregnancy, with a prevalence of 2 per 100,000 hospital admissions, the risk of recurrent VT in patients with congenital heart disease is high, approximately 27% of cases [2]. VAs most commonly occur in the setting of congenital heart disease, nonischemic or ischemic cardiomyopathies, inherited arrhythmia syndromes, or QT prolongation due to drugs or electrolyte abnormalities [1]. In the absence of structural heart disease, VA is typically hemodynamically stable and associated with a good prognosis [111,113].

In cases of hemodynamic instability, electrical synchronized cardioversion should be performed urgently due to the high risk of fetal compromise. Electrical synchronized cardioversion at 50–100 J (and if needed, higher energies at 100–360 J) can be performed [62].

Lidocaine is the first-line option for stable patients with ventricular arrhythmias during pregnancy [62]. If lidocaine is ineffective, procainamide or quinidine can be considered as alternatives. Amiodarone is reserved for life-threatening situations when other therapies have failed [114]. Magnesium can be safely used for torsade de pointes or polymorphic ventricular tachycardia, administering 1–2 g intravenously [62].

Case reports suggest successful ablation in some cases, but it is generally considered an option of last resort, with the procedure deferred to the postpartum period [115,116].

Pregnancy in patients with inherited arrhythmia syndromes is generally well-tolerated. The evaluation for each specific syndrome involves risk stratification, the assessment of potential triggers during the peripartum period, and appropriate pharmacologic therapy [2]. Long QT syndrome stands out as the most common inherited arrhythmia syndrome in pregnant women. Other less common syndromes comprise catecholaminergic polymorphic ventricular tachycardia, Brugada syndrome, and arrhythmogenic right ventricular cardiomyopathy.

In the *long QT syndrome*, the risk of arrhythmic events is not increased [117,118].

In type 1, the critical phase is during labor and delivery, where the adrenergic triggers play an arrhythmogenic role [119]. In type 2, auditory stimuli and loud noises could be the arrhythmogenic triggers. Attention should be paid to other QT-prolonging medications, including anti-emetics such as ondansetron. Beta-blockers are highly effective in preventing arrhythmic events and are recommended for continuous use in all pregnant patients with long QT syndrome, especially during the high-risk postpartum period [9,118]. Propranolol (nonselective beta-blocker) is preferred due to its extensive safety record and if ventricular tachycardia has already been treated with nadolol, the drug can be continued during pregnancy. On the other hand, atenolol is the only medication that should not be administered because of its potentially greater risk of fetal adverse events.

Physical exertion and the emotional stress of labor and delivery can trigger a *ventricular tachycardia* named *catecholaminergic polymorphic*. Nonselective beta-blockers play a key role in management as well as the continuous use of beta-blockers throughout pregnancy and the postpartum period. Flecainide may be introduced as synergism (less as second choice) if events persist despite beta-blocker therapy [119].

For *Brugada syndrome*, more prevalent in men, there are limited data on management during pregnancy. Arrhythmic events in this syndrome usually occur during periods of high vagal tone. The use of quinidine has shown effectiveness in reducing arrhythmic events during pregnancy [111].

Pregnancy in women with *arrhythmogenic right ventricular cardiomyopathy* is generally safe. The continuation of beta-blockers during pregnancy, especially in patients with a history of ventricular arrhythmias, is recommended [2].

*Pregnancy-associated spontaneous coronary artery dissection (P-SCAD)* is internal tearing or acute bleeding within the tunica media of the arterial wall not resulting from trauma. It occurs in patients younger than 50 years with acute manifestations of acute myocardial infarction (MI) and cardiogenic shock. Pregnancy-related SCAD occurs in the first 12 weeks postpartum while non-pregnancy-associated spontaneous coronary artery dissection (NP-SCAD) can happen in any other period of a woman’s life.

Fibromuscular dysplasia and systemic inflammatory conditions are the most common conditions associated with NP-SCAD while connective tissue disorder is related to P-SCAD and additional care is required when treating women that have a history of multiple births and preeclampsia. The lab findings are very high troponin levels (>500× upper limit of normal) and ST-segment elevation myocardial infarction.

Noninvasive treatment is recommended for stable patients and PCI is recommended only for those patients with poor coronary flow, persistent chest pain, persistent ST elevation, and hemodynamic instability. Moreover, P-SCAD patients have a greater rate of complications after the procedure, such as repeat PCI, CABG, cardiogenic shock, and maternal death. Pharmacological treatment is similar to that adopted for ACS patients [116,120].

### 5.6. Bradyarrhythmias

Bradyarrhythmias are uncommon in pregnancy because pregnant women are not predisposed to high-degree atrioventricular blocks [1]. However, women with repaired congenital heart disease or prior cardiac surgery are at an increased risk for bradyarrhythmias.

Bradyarrhythmias, if present, are frequently identified prior to pregnancy. It has been observed that women with untreated atrioventricular block are more prone to experience progression in conduction disease during pregnancy [1]. In cases where there is a need for device implantation during pregnancy, the procedure can be performed safely with minimal fluoroscopy and under echocardiographic guidance [2]. This approach helps to mitigate potential risks associated with radiation exposure while ensuring the well-being of both the mother and the developing fetus.

### 5.7. Cardiac Arrest in Pregnancy

While cardiac arrest in pregnant women can be caused by various factors, including hemorrhage, pulmonary embolism, sepsis, preeclampsia/eclampsia, ictus, amniotic fluid embolism and anesthetic complications (e.g., failed intubation, local anesthetic toxicity, aspiration, high neuraxial block), cardiovascular causes should also be considered [120]. Common cardiovascular causes include heart failure, acute myocardial infarction, aortic dissection, pulmonary edema, and arrhythmias [4].

It is noteworthy that during pregnancy the underlying causes of cardiac arrest are often reversible, such as hemorrhage. Additionally, hormonal changes during pregnancy may enhance myocardial and cerebral blood flow during cardiopulmonary resuscitation. These factors contribute to better outcomes of cardiac arrest in pregnant women compared to nonpregnant women.

The distinctive aspects of managing cardiac arrest in pregnant women are summarized in Table 3. In particular, early emergency cesarean delivery, also referred to as resuscitative hysterotomy, holds the potential to be a life-saving intervention for both the mother and fetus. This approach is considered a viable option for pregnancies at/or beyond 20 weeks of gestation, aiming to alleviate aortocaval compression and facilitate the restoration of spontaneous circulation, irrespective of the fetal condition. Despite the appropriate implementation of leftward uterine displacement, the mechanical impact of the gravid uterus can lead to a reduction in venous return from the inferior vena cava, obstruction of blood flow through the abdominal aorta, and a decrease in thoracic compliance. These factors collectively contribute to the challenges encountered in achieving successful CPR. Furthermore, it is noteworthy that emergency cesarean delivery performed beyond 22 weeks of gestation may yield neonatal benefits for the newborn [115,116,117,118,119,120].

## 6. Conclusions

Arrhythmias represent the predominant cardiovascular complication during pregnancy, manifesting as either new-onset or exacerbation of preexisting conditions. Optimal management necessitates prenatal counseling emphasizing the heightened risk of arrhythmia recurrence during gestation. Consideration of ablation procedures prior to pregnancy and vigilant monitoring in high-risk cohorts are essential to ensure favorable maternal and fetal outcomes. A comprehensive multidisciplinary approach for arrhythmia management during pregnancy is an important requisite, extending through the phases of labor and delivery.

It is important to stress that cardiac monitoring must be reliable and continuous because the best therapy for pregnant women and their fetus or newborn is prevention. 

## Figures and Tables

**Table 1:** Antiarrhythmic drugs and their safety profiles and adverse events in pregnancy and lactation.

AR Drug	FDA Category	Fetal Adverse Effect	Use in Pregnancy	Lactation
**Adenosine**	C	No significant adverse events. Fetal monitoring for transient fetal bradycardia	Safe	Safe
**Amiodarone**	D	Fetal hypothyroidism with congenital goiter, growth retardation, preterm birth, and neurodevelopmental abnormalities	Only if other options are not available	Do not use
**Atenolol**	D	Increased risk of congenital malformations, FGR, and fetal bradycardia	Not safe	Do not use
**Atropine**	C	Scant literature regarding the safety profile	Used for emergent resuscitation	Not safe
**Diltiazem**	C	Increased risk of FGR. Skeletal, cardiac, tongue, and retinal abnormalities in animal models	Use with caution	Use with caution
**Digoxin**	C	No significant adverse effects	Safe	Safe
**Dofetilide**	C	In animal models, fetal resorption and skeletal abnormalities have been observed if administered during organogenesis, and significant bradycardia even at the lower doses	Do not use	Do not use
**Dronedarone**	X	Significant fetal adverse events including vascular and limb abnormalities and cleft palate	Do not use	Do not use
**Flecainide**	C	Animal data have confirmed that it is free from teratogenic effects. At very high doses, delayed sternal and vertebral ossification observed in rats	Use with caution	Safe
**Ibutilide**	C	No adverse events reported in case reports. In rats, skeletal and cardiac abnormalities noted with daily exposure	Use with caution	Use with caution
**Ivabradine**	NA	High incidence of fetal cardiac defects in rats and ectrodactyly in rabbits. FGR, neonatal bradycardia, and hypotension can occur	Do not use	Do not use
**Lidocaine**	B	No increased risk of birth defects or adverse events have been observed at therapeutic doses	Safe	Safe
**Metoprolol**	C	No significant adverse effects. Small risk of FGR, neonatal bradycardia, and hypoglycemia	Use with caution	Safe
**Mexiletine**	C	Minimal data. Concern for lower Apgar scores. Increased fetal resorption at 4× maximum RHD in rats and rabbits	Use with caution	Use with caution
**Nadolol**	C	Small risk of apnea, FGR, and hypoglycemia	Use with caution	Do not use
**Procainamide**	C	No teratogenic effects, limited data reported	Use with caution	Use with caution
**Propafenone**	C	Minimal human data. Reduction in neonatal survival, weight gain, and development abnormalities observed at 3–6× maximum RHD	Use with caution	Do not use
**Propranolol**	C	No significant adverse effects. Small risk of FGR, neonatal bradycardia, and hypoglycemia	Use with caution	Safe
**Quinidine**	C	No teratogenicity observed. Rarely, mild uterine contractions, premature labor, neonatal thrombocytopenia, and cranial nerve VIII damage have been reported	Use with caution	Use with caution
**Sotalol**	C	No teratogenic potential. Small risk of fetal bradycardia and hypoglycemia	Safe	Use with caution
**Verapamil**	C	No significant teratogenicity risk, only maternal hypotension and fetal bradycardia	Use with caution	Use with caution

FGR: fetal growth restriction; RHD: recommended human dose.

**Table 2:** Arrhythmias and treatment during pregnancy.

**ATRIAL FIBRILLATION (AF)**			
**Patient Hemodynamically Stable**	Patient Hemodynamically Unstable *		**Recurrent AF**
*Obtain rate control*:	Electrical synchronized cardioversion		Flecainide or sotalol
1st line: beta-blockers +/− digoxin			
2nd line: ca-channel blockers			
In case of inadequate rate control: electrical synchronized cardioversion			If refractory, consider ablation with minimal fluoroscopy
**AF persisting for more than 48 h should be managed with anticoagulation before cardioversion**			
**SUPRAVENTRICULAR TACHYCARDIA (SVT)**			
***Patient hemodynamically stable***	Patient hemodynamically unstable *		***Recurrent SVT***
1st line: vagal maneuvers (Valsalva maneuver or carotid sinus massage)	Electrical synchronized cardioversion		1st line: beta-blockers +/− digoxin in patients without pre-excitation
2nd line: adenosine			2nd line: ca-channel blockers
			In patients with pre-excitation: beta-blockers + flecainide
			If refractory consider ablation with minimal fluoroscopy
**VENTRICULAR TACHYCARDIA (VT)**			
**Patient hemodynamically stable**	Patient hemodynamically unstable *	**Polymorphic VT or torsade de pointes**	**Long-QT syndorme**
1st line: lidocaine	Electrical synchronized cardioversion	Magnesium sulfate	*Avoid QT-prolonging medications*
2nd line: procainamide or quinidine Amiodarone is reserved for life-threatening situations when other options have failed			Propranolol prevents arrhythmic events
		
Monomorphic ventricular tachycardia: ablation with minimal fluoroscopy only if refractory			

* After 20 weeks of gestation, place the patient in the left lateral tilt position to prevent aortocaval compression.

**Table 3:** Management of cardiac arrest in pregnancy.

Point	Recommendation/Action
Multidisciplinary Team	Ensure a multidisciplinary team, including anesthesiologists, cardiologists, obstetricians, and neonatologists, for optimal maternal and fetal care during cardiac arrest.
Chest Compressions	Perform chest compressions in the standard position on the sternum.
Intravenous Access	Place intravenous access above the diaphragm to ensure drug efficacy, as femoral administration may not reach the maternal heart until fetal delivery.
Medication Administration	Do not withhold medications due to concerns for fetal teratogenicity, including amiodarone.
Drug Doses and Defibrillation Protocols	Maintain standard drug doses and defibrillation protocols during resuscitation efforts.
Fetal Monitoring	Interrupt fetal monitoring and remove equipment during defibrillation to prevent electrocution injury to the patient or rescuers.
Airway Management	Assume a difficult airway. Intubation via video-laryngoscopy using a smaller-sized endotracheal tube is recommended; consider supraglottic airway devices if intubation is challenging.
Gestational Age Estimation	Estimate gestational age, taking into account the common occurrence of aorto-caval compression after 20 weeks. Additionally, acknowledge that neonates born at 23 and 24 weeks have demonstrated a reasonable chance of survival without severe deficits. When prenatal records are unavailable, rely on physical examination, considering key indicators. Notably, by 20 weeks in a singleton pregnancy, the top of the uterine fundus is typically aligned with the umbilicus.
Uterine Displacement	Manually displace the uterus laterally and to the left (left uterine displacement) for pregnancies ≥20 weeks to avoid aorto-caval compression. If manual displacement is not possible, tilt the operating table or use towels/blankets to achieve a tilt of no more than 30°.
Emergency Cesarean Delivery Preparation	Prepare for early emergency caesarean delivery for pregnancies ≥20 weeks.
Cesarean Delivery Timing	If spontaneous circulation is not restored within five minutes of maternal cardiac arrest, initiate emergency caesarean delivery at 20 weeks of gestation and beyond.
Extracorporeal Life Support Evaluation	Evaluate the need for instituting extracorporeal life support based on the clinical scenario.

## Data Availability

Not applicable.
